# Detailed Analysis of Intraretinal Layers and Their Correlation to Brain Structures in Normal Healthy Aging

**DOI:** 10.1167/tvst.15.10.1

**Published:** 2026-10-05

**Authors:** Jack Anthony Cipolla, Giovana Rosa Gameiro, William Tackett, Jessica Morgan, Geoffrey Aguirre, Pradip Pattany, Robert O'Brien, Hong Jiang, John A. Detre, Jianhua Wang

**Affiliations:** 1 University of Miami Miller School of Medicine, Miami, FL, USA; 2 Department of Ophthalmology, Bascom Palmer Eye Institute, University of Miami Miller School of Medicine, Miami, FL, USA; 3 Department of Ophthalmology and Visual Sciences, Federal University of Sao Paulo – EPM/UNIFESP, Sao Paulo, SP, Brazil; 4 Department of Neurology, University of Pennsylvania Perelman School of Medicine, Philadelphia, PA, USA; 5 Department of Radiology, University of Miami Miller School of Medicine, Miami, FL, USA; 6 Biostatistics Center, Bascom Palmer Eye Institute, University of Miami Miller School of Medicine, Miami, FL, USA; 7 Department of Neurology, University of Miami Miller School of Medicine, Miami, FL, USA

**Keywords:** aging, retinal tissue volume, visual cortex, optical coherence tomography (OCT), magnetic resonance imaging (MRI)

## Abstract

**Purpose:**

The purpose of this study was to investigate the relationships between intraretinal layer volumes and visual cortical thickness in healthy individuals and determine the influence of age on these associations.

**Methods:**

Fifty-three subjects were recruited. One eye per subject was imaged using optical coherence tomography (OCT) to quantify volumes of total retinal thickness (TRT), retinal nerve fiber layer (RNFL), ganglion cell–inner plexiform layer (GCIPL), inner nuclear layer (INL), outer nuclear layer (ONL), outer plexiform layer (OPL), and photoreceptor (PR) layer. Brain magnetic resonance imaging (MRI) was performed, and cortical thickness was quantified in 13 visual cortex regions using the Schaefer 2018 atlas.

**Results:**

TRT, RNFL, and GCIPL volumes were significantly correlated with cortical thickness in multiple visual regions (*r* = 0.34–0.59, all *P* < 0.05). These associations were largely attenuated after adjusting for age. In contrast, selected RNFL and INL associations remained significant after age adjustment. OPL, ONL, and PR layer showed no significant correlations with cortical thickness. PR layer volume increased with age (*r* = 0.38, *P* < 0.01), unlike the age-related thinning observed in other layers.

**Conclusions:**

Distinct retinal layers exhibit differential relationships with the visual cortex, with some associations predominantly mediated by age and selected RNFL and INL associations persisting independently of age.

**Translational Relevance:**

Distinguishing age-mediated from age-independent retinal–cortical relationships may help identify OCT-derived retinal measures that differentiate normal aging from cerebral pathology and support their investigation as noninvasive biomarkers of central nervous system (CNS) integrity.

## Introduction

The retina, as an extension of the central nervous system (CNS), offers a noninvasive and anatomically accessible means of assessing brain structure and function. Sharing embryologic origins and neuronal architecture with the brain, the retina provides a potential biomarker for cerebral pathology and aging-related degeneration.^1^^–^^3^ Optical coherence tomography (OCT) enables high-resolution imaging of retinal layers and has been increasingly used to study neurodegenerative diseases such as Alzheimer's disease (AD), Parkinson's disease, and multiple sclerosis.^4^^–^^7^ However, structural retinal changes also occur as part of the normal aging process, and their relationship to cerebral alterations remains incompletely understood.

Aging is associated with widespread cortical thinning and reductions in total brain volume, particularly in occipital and parietal regions involved in visual processing, as well as an expansion of the ventricular system.^8^^,^^9^ Simultaneously, OCT studies have documented age-related decreases in total retinal thickness and in specific inner retinal layers, including the ganglion cell–inner plexiform layer (GCIPL) and retinal nerve fiber layer (RNFL), which reflect neuronal loss.^10^^,^^11^ Although individual studies have reported associations between global retinal thickness and brain morphology, few have used detailed segmentation of both intraretinal layers and cortical regions to identify layer-specific, region-specific relationships.^12^^,^^13^

Recent large-scale imaging studies have provided valuable insights into this connection.^14^ Notably, Chua et al., using data from the UK Biobank, demonstrated that thinner macular retinal layers—particularly the GCIPL—were significantly associated with reduced total brain volume and occipital lobe volume in older adults.^15^ Similarly, Sun et al. identified structural correlations between inner retinal measures, such as the RNFL and GCIPL, and magnetic resonance imaging (MRI)-derived cortical and subcortical brain volumes, with notable associations in the occipital cortex and cerebellum.^16^ Extending these findings to a younger population, Rebouças et al. reported associations between RNFL thickness and the integrity of the visual white matter tracts in healthy young adults, suggesting that retinal and brain structures may reflect common developmental or functional pathways even in early adulthood.^17^ Additional studies have extended these findings to cognitively normal adults at risk for AD or systemic diseases such as diabetes, further highlighting the retina's potential as a cerebral biomarker.^18^^,^^19^

Despite this growing body of evidence, most prior studies have examined global retinal or brain metrics, lacked layer-level granularity, or did not control for age in assessing structural correlations. To address these limitations, it is crucial to investigate whether specific intraretinal layers correlate with cortical thickness in visual brain regions. By evaluating both unadjusted and age-adjusted associations in a cohort of healthy individuals, age-mediated and age-independent relationships between retinal and brain structures can be explored. This approach offers new insights into the parallel aging processes of the retina and brain, highlighting the possible role of intraretinal layers as markers for cerebral structural integrity during aging. Therefore, the goal of the present study was to investigate the correlations between retinal and brain structures in healthy individuals.

## Methods

### Subjects

The research project was approved by the Institutional Review Board (IRB) for Human Research at the University of Miami. A detailed description of the study was provided to each participant, who then read and signed an IRB-approved written informed consent form. The tenets of the Declaration of Helsinki were observed throughout the study.

A screening visit was conducted to confirm participant eligibility. Exclusion criteria included pre-existing neurological conditions such as AD or mild cognitive impairment, as well as ocular conditions that may affect macular thickness, including macular degeneration, glaucoma, and high myopia, defined as refractive errors exceeding –6.0 diopters. Other systemic comorbidities, such as hypertension, diabetes, or hyperlipidemia, were not exclusionary. A total of 53 participants (26 men and 27 women, age range = 21–80 years, mean ± SD: 47.5 ± 20.6 years) were enrolled.

### Spectral Domain-OCT Measurements

Each participant underwent spectral-domain OCT (SD-OCT) imaging using the Optovue OCTA system (Angiovue; Optovue, Inc., Fremont, CA; software version 2018.1.0.43), as described in a previous study.^20^ The system combines real-time eye tracking and DualTrac Motion Correction Technology (MCT) to reduce motion artifacts. Eye tracking uses infrared fundus imaging to detect involuntary movements and adjust scanning in real time. MCT then registers and merges two orthogonal scan volumes (Fast-X and Fast-Y) through pixel-level alignment, enhancing image quality.^21^

Imaging was performed in one eye using a 6 × 6 mm macular scan centered on the fovea. The scan protocol consisted of 400 B-scans per volume, each composed of 400 A-scans, yielding volumetric imaging of the macular region. The device acquires data at a scan speed of 70,000 A-scans per second, with an axial resolution of approximately 5 µm.^20^^,^^22^

Each subject underwent 1 scan per session, and only images with a quality score ≥7/10 were included in the analysis. All OCT images were visually inspected for motion artifacts, blink-related distortions, and overall image quality. When image quality was deemed insufficient, additional scans were obtained at the time of acquisition.

The OCT data were segmented into seven intraretinal layers using commercially available software (Orion; Voxeleron LLC, Pleasanton, CA). Thickness and volume measurements were obtained for total retinal thickness (TRT), RNFL, GCIPL, inner nuclear layer (INL), outer plexiform layer (OPL), outer nuclear layer (ONL), and photoreceptor (PR) layer (Fig. 1). Retinal tissue volume (RTV) measurements were obtained from a perifoveal disk centered on the fovea, defined by an outer diameter of 6 mm. Researchers reviewed the exported segmented whole-retina thickness maps and the horizontal central B-scan to assess segmentation accuracy and identify any abnormalities. Volumetric data from these layers were used in subsequent correlation analyses.

**Figure 1.**
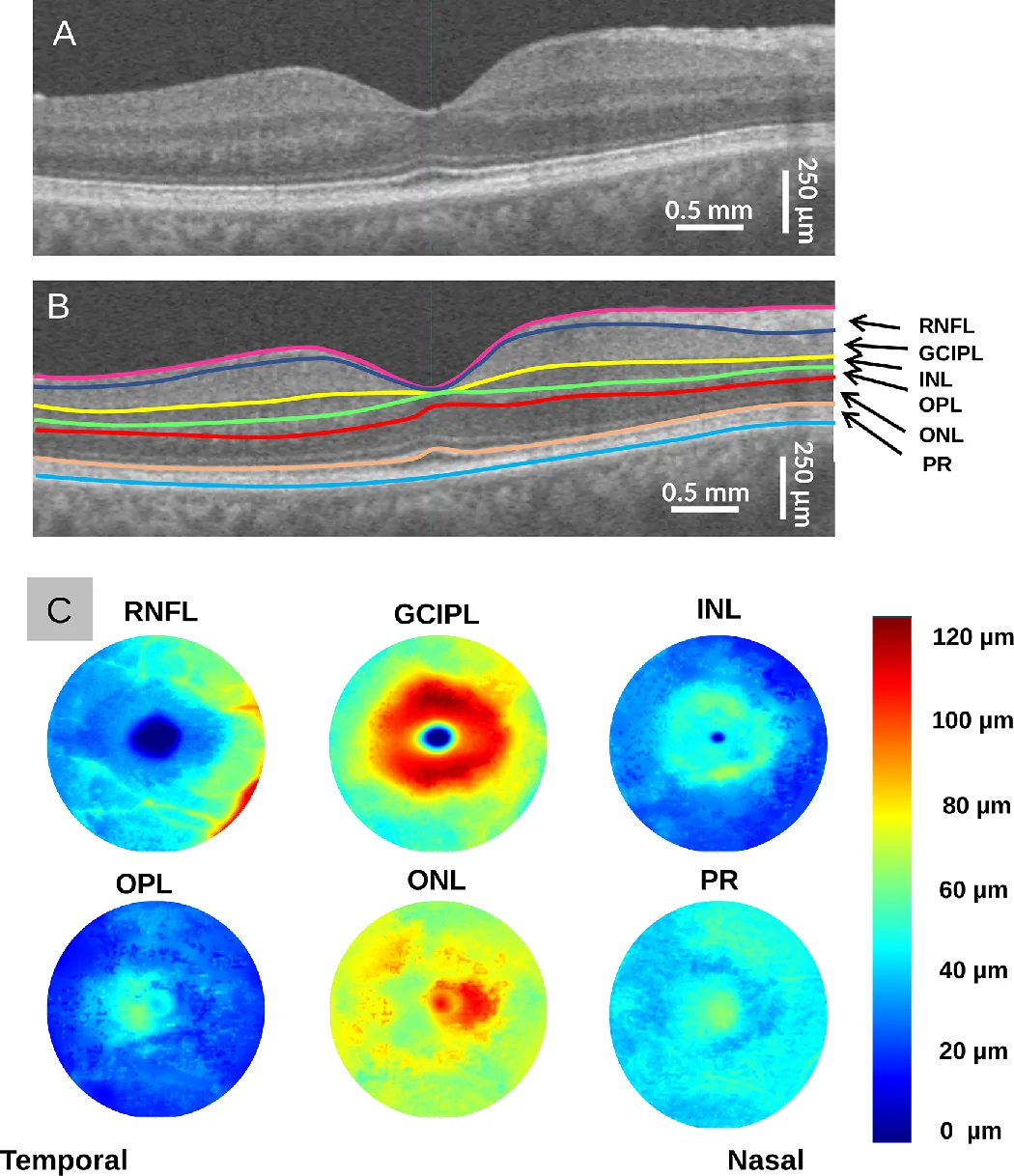
Cross-sectional retina and segmented tomographic thickness maps of intraretinal layers Optical coherence tomography (OCT) was used to scan a healthy subject’s retina (**A**). Six intraretinal layers (**B**) were segmented with the Orion software. There are six intraretinal layers, defined by seven segmented boundaries, which correspond to six tomographic thickness maps (**C**, diameter of 6 mm). RNFL, retinal nerve fiber layer; GCIPL, ganglion cell–inner plexiform layer; INL, inner nuclear layer; ONL, outer nuclear layer; OPL, outer plexiform layer; and PR, retinal photoreceptor layer.

### MRI Brain Imaging

Each subject underwent brain MRI, and a total of 200 different brain measurements were obtained with MRI. The MRI was carried out using a 3 Tesla MRI system (GE 750 Discovery system). The MRI protocol consisted of a three-axis localizer T1-weighted structural MRI (TR/TE/TI = 2400/2.24/1060 msec acquired at 0.8 mm^3^ isotropic resolution using rate = 2 GRAPPA/ARC acceleration; HCP protocol).^23^^,^^24^ High-resolution T1-weighted MRI scans were processed using validated surface-based and diffeomorphic pipelines (FreeSurfer and Advanced Normalization Tools [ANTs]), which perform tissue classification, cortical surface reconstruction, and cortical thickness estimation.^25^^,^^26^ Cortical thickness was defined as the distance between the gray–white matter boundary and the pial surface, a well-established marker of cortical integrity.^27^

Following cortical reconstruction, the cerebral cortex was parcellated into functionally defined regions based on intrinsic resting-state functional connectivity. This approach builds on large-scale cortical network organization originally described by Yeo et al.^24^ We used the Schaefer 2018 atlas, which refines this framework into 200 spatially localized cortical parcels across 17 functional networks, preserving network-level organization and functional coherence (Fig. 2).^28^

**Figure 2.**
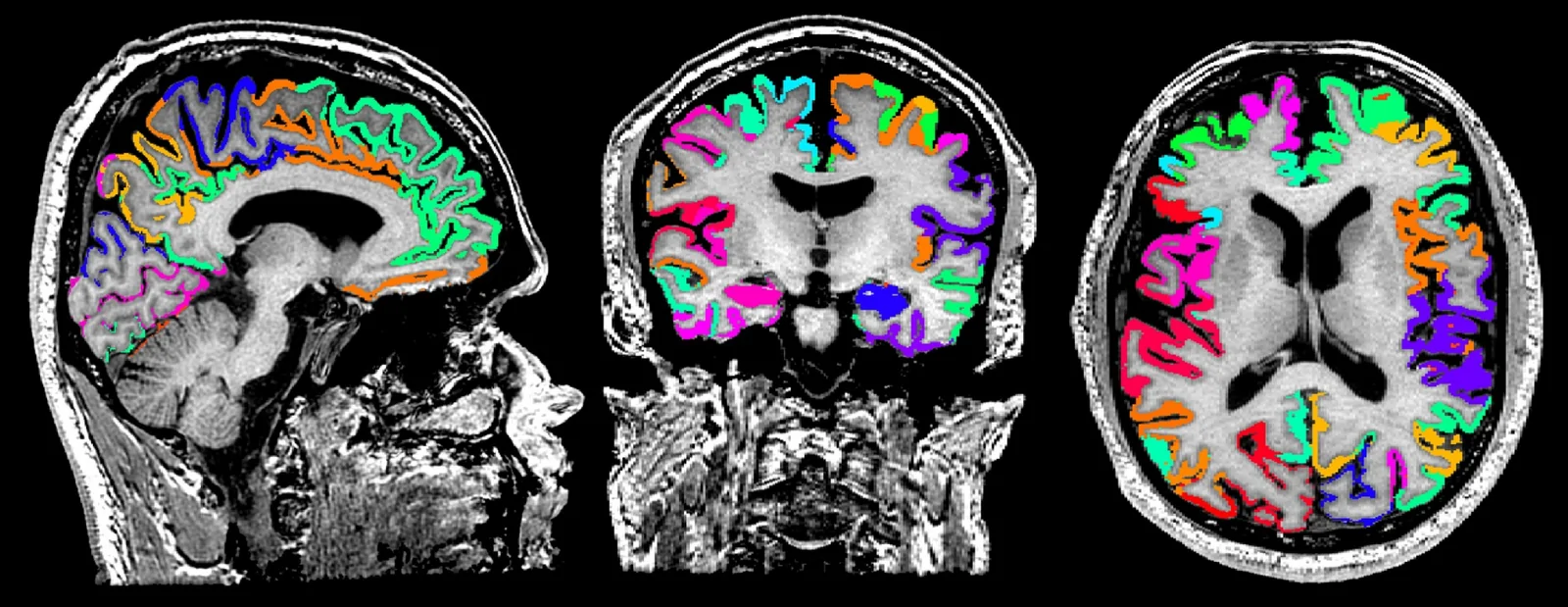
Sagittal, coronal, and axial T1-weighted MRI images demonstrating cortical parcellation using the Schaefer 2018 atlas (200 parcels, 17 networks). Color-coded regions represent cortical parcels used for cortical thickness analysis.

Recent work has further demonstrated that such areal-level parcellations can be reliably estimated at the individual level using hierarchical Bayesian models, yielding parcels that are spatially localized, reproducible across sessions, and behaviorally meaningful.^23^ Regions corresponding to the visual cortex were extracted bilaterally, and cortical thickness values were averaged across hemispheres for subsequent analyses and correlation with retinal layer measurements.

### Statistical Analysis

All statistical analyses were conducted using IBM SPSS Statistics (version 27; IBM Corp., Armonk, NY) and R software program (The R Foundation for Statistical Computing, Vienna, Austria). To avoid potential bias from inter-eye correlations, only one eye per subject was included in the analysis, with a preference for the right eye when available. Regional cortical thickness values from the left and right hemispheres were averaged to account for anatomic symmetry and to reduce the number of comparisons, thereby improving statistical power and interpretability.

Pearson correlation coefficients were used to assess associations between retinal layer volumes and cortical thickness across visual brain regions. Normality of variables was assessed using the Shapiro-Wilk test. To evaluate age-independent relationships, partial correlations controlling for age were also computed. Multiple comparisons were corrected using the Benjamini-Hochberg procedure to control the false discovery rate (FDR), as described by Benjamini and Hochberg.^29^ This correction was applied separately for each set of comparisons involving retinal layers and brain regions. Correlations among retinal layers and among brain regions were also examined using the same statistical framework. Statistical significance was set at an adjusted *P* value of < 0.05.

## Results

All 13 visual cortex regions were included in the correlation analysis and are reported in Table 1. Partial correlations controlling for age are presented in Table 2. For visualization purposes, the five visual cortex regions showing correlations with the largest absolute magnitude for each retinal layer are highlighted in the figures. This approach captures the strongest associations, regardless of direction, allowing both positive and negative correlations to be represented.

**Table 1.** Correlations of the 13 Visual Cortex Regions With the Seven Retinal Layers and With Age

Brain Region/Retinal Layer	TRT	RNFL	GCIPL	INL	OPL	ONL	PR	Age
Central Visual Extrastriate 2	0.55^b^	0.57^b^	0.55^b^	0.31	0.17	0.17	−0.14	−0.79^b^
Central Visual Striate 1	0.53^b^	0.59^b^	0.34^a^	0.35^a^	0.12	0.30	0.11	−0.39^a^
Peripheral Visual Inferior Extrastriate 2	0.51^b^	0.51^b^	0.55^b^	0.35^a^	0.12	0.15	−0.24	−0.78^b^
Peripheral Visual Superior Extrastriate 1	0.48^b^	0.40^a^	0.56^b^	0.23	0.19	0.16	−0.24	−0.81^b^
Peripheral Visual Superior Extrastriate 2	0.47^b^	0.46^b^	0.50^b^	0.25	0.07	0.24	−0.21	−0.76^b^
Peripheral Visual Striate Calcarine 1	0.47^b^	0.51^b^	0.47^b^	0.32	0.03	0.24	−0.23	−0.75^b^
Central Visual Extrastriate 1	0.43^b^	0.53^b^	0.45^b^	0.20	0.14	0.08	−0.15	−0.76^b^
Peripheral Visual Inferior Extrastriate 1	0.43^b^	0.57^b^	0.50^b^	0.19	0.08	0.06	−0.26	−0.80^b^
Central Visual Extrastriate 3	0.42^b^	0.50^b^	0.43^b^	0.20	0.09	0.12	−0.13	−0.69^b^
Central Visual Extrastriate 5	0.40^a^	0.49^b^	0.46^a^	0.20	0.05	0.11	−0.21	−0.69^b^
Peripheral Visual Superior Extrastriate 3	0.39^a^	0.45^b^	0.47^b^	0.09	0.13	0.10	−0.27	−0.75^b^
Peripheral Visual Inferior Extrastriate 3	0.37^a^	0.29	0.41^a^	0.32	0.24	0.02	−0.12	−0.51^b^
Central Visual Extrastriate 4	0.29	0.31	0.35^a^	0.08	0.12	0.04	−0.17	−0.69^b^

a Denotes adjusted *P* < 0.05.

b Denotes adjusted *P* < 0.01.

**Table 2.** Partial Correlations Between all 13 Visual Cortex Regions and the 7 Retinal Layers Controlling for Age

Brain Region/Retinal Layer	TRT	RNFL	GCIPL	INL	OPL	ONL	PR
Central Visual Extrastriate 2	0.29	0.21	0.09	0.30	0.10	0.16	0.28
Central Visual Striate 1	0.43**	0.49**	0.12	0.31	0.07	0.29	0.30
Peripheral Visual Inferior Extrastriate 2	0.20	0.10	0.10	0.36*	0.02	0.13	0.10
Peripheral Visual Superior Extrastriate 1	0.14	−0.15	0.08	0.18	0.13	0.16	0.11
Peripheral Visual Superior Extrastriate 2	0.14	0.02	−0.01	0.20	−0.05	0.26	0.13
Peripheral Visual Striate Calcarine 1	0.15	0.12	−0.05	0.30	−0.11	0.27	0.08
Central Visual Extrastriate 1	0.08	0.16	−0.10	0.12	0.06	0.03	0.22
Peripheral Visual Inferior Extrastriate 1	0.04	0.21	−0.06	0.11	−0.06	−0.02	0.08
Central Visual Extrastriate 3	0.11	0.16	−0.04	0.12	−0.01	0.08	0.19
Central Visual Extrastriate 5	0.09	0.14	0.01	0.12	−0.06	0.06	0.08
Peripheral Visual Superior Extrastriate 3	0.01	0.02	−0.04	−0.05	0.04	0.05	0.03
Peripheral Visual Inferior Extrastriate 3	0.15	−0.01	0.12	0.28	0.20	−0.03	0.09
Central Visual Extrastriate 4	−0.11	−0.17	−0.18	−0.05	0.03	−0.02	0.14

* Denotes adjusted *P* < 0.05.

** denotes adjusted *P* < 0.01.

To provide context for the correlation analyses and facilitate comparison with prior studies, the mean and standard deviation (SD) of TRT and individual intraretinal layer volumes across all participants are summarized in Table 3. TRT averaged 7.94 ± 0.38 mm³, with inner retinal layers demonstrating mean volumes of 1.15 ± 0.11 mm³ for RNFL, 1.93 ± 0.20 mm³ for GCIPL, 0.94 ± 0.05 mm³ for INL, and 0.67 ± 0.08 mm³ for OPL. Outer retinal layers included an ONL volume of 2.06 ± 0.12 mm³ and a PR layer volume of 1.17 ± 0.08 mm³.

**Table 3.** Mean ± SD of Retinal Layer Volumes in Healthy Participants

Retinal Layer	Volume (mm³) Mean ± SD
TRT	7.94 ± 0.38
RNFL	1.15 ± 0.11
GCIPL	1.93 ± 0.20
INL	0.94 ± 0.05
OPL	0.67 ± 0.08
ONL	2.06 ± 0.12
PR	1.17 ± 0.08

GCIPL, ganglion cell–inner plexiform layer; INL, inner nuclear layer; ONL, outer nuclear layer; OPL, outer plexiform layer; PR, photoreceptor layer; RNFL, retinal nerve fiber layer; TRT, total retinal thickness.

Significant positive correlations (adjusted *P* < 0.01 or *P* < 0.05) were observed among several visual cortex regions and specific retinal layers. Most notably, TRT, RNFL, and GCIPL volumes showed consistently strong correlations across nearly all visual cortex regions. For example, the central visual extrastriate two region demonstrated robust positive correlations with TRT (*r* = 0.55), RNFL (*r* = 0.57), and GCIPL (*r* = 0.55), all significant at *P* < 0.01. Similarly strong correlations were found in peripheral regions, including the peripheral visual inferior extrastriate 2 and superior extrastriate 1, among others.

In contrast, the PR layer, OPL, and ONL did not show any significant associations with visual cortex regions, suggesting a weaker structural link between these outer retinal layers and cortical anatomy. Interestingly, the PR layer demonstrated a positive correlation with age (*r* = 0.38, *P* < 0.01), which contrasts with the general pattern of age-related decline observed in the other retinal layers. This divergent trend is further illustrated in Figure 3.

**Figure 3.**
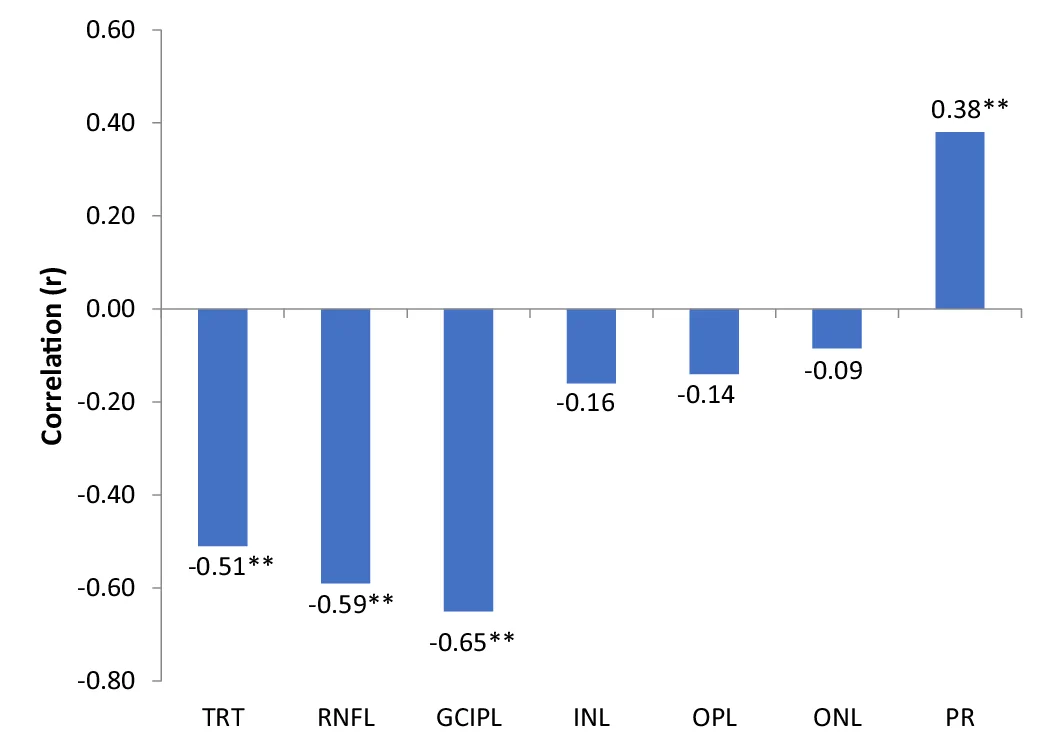
Correlation between retinal layer volume and age. The table presents Pearson correlation coefficients (*r* values) for total retinal thickness (TRT) and individual retinal layers: RNFL (retinal nerve fiber layer), GCIPL (ganglion cell–inner plexiform layer), INL (inner nuclear layer), OPL (outer plexiform layer), ONL (outer nuclear layer), and PR (photoreceptor layer). The TRT, RNFL, and GCIPL exhibited a significant negative correlation with age (*P* < 0.01), indicating age-related thinning in these layers. The PR layer, in contrast, showed a positive correlation with age (*P* < 0.01).

Consistent with these findings, age was significantly negatively correlated with all visual cortex regions (*P* < 0.05 or *P* < 0.01), with the most pronounced decline observed in peripheral visual superior extrastriate 1 (*r* = –0.81, *P* < 0.01).

TRT was significantly correlated with central visual extrastriate 2, central visual striate 1, peripheral visual inferior extrastriate 2, and peripheral visual superior extrastriate 1 and 2 before age adjustment (*P* < 0.01; Fig. 4A). After controlling for age, only the correlation with central visual striate 1 remained significant (*r* = 0.43, *P* < 0.01), suggesting age influences most of these relationships.

**Figure 4.**
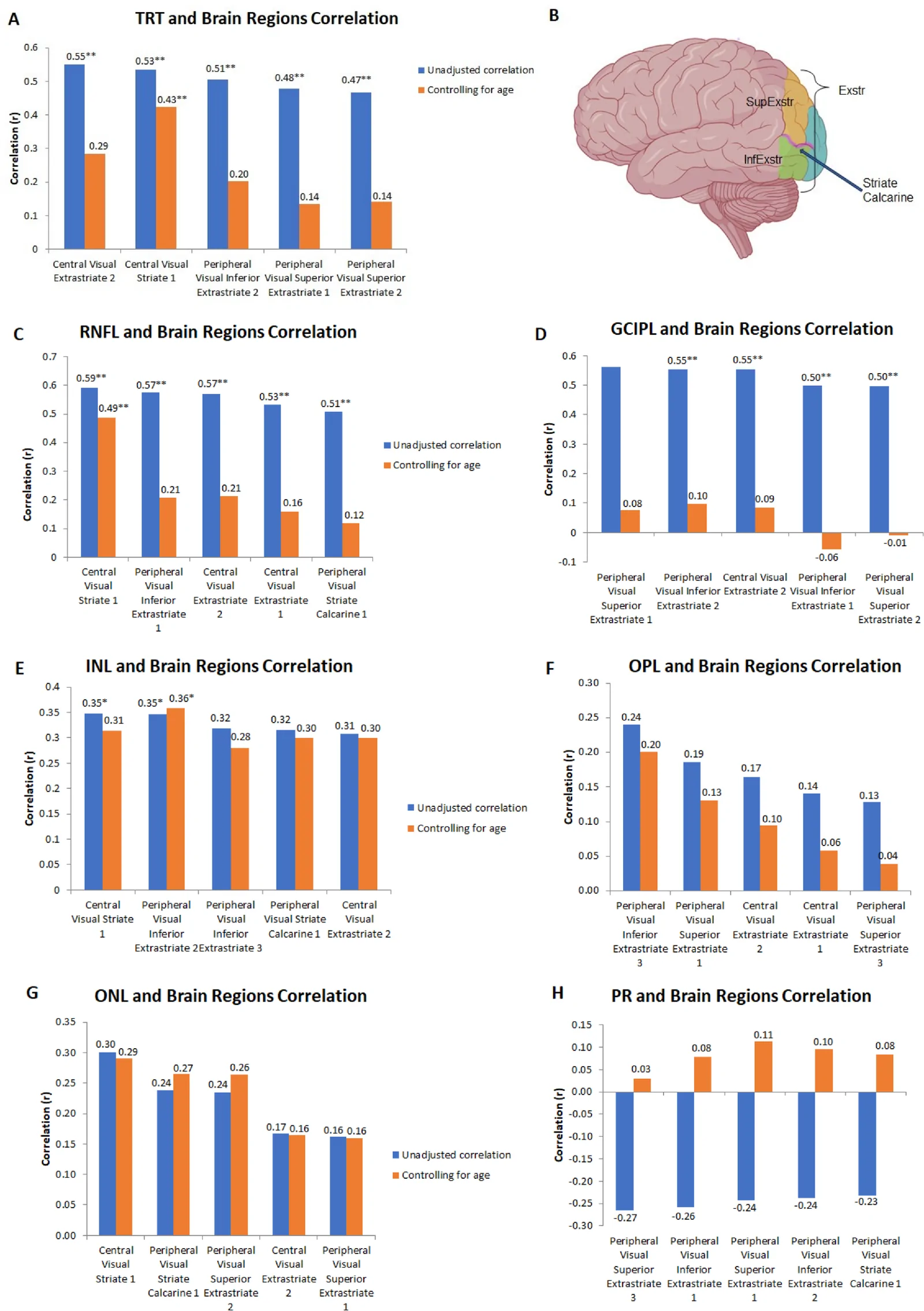
Correlations between intraretinal layer volumes and visual cortex regions in healthy aging. (**A**) shows the correlations between total retinal thickness (TRT) and the five visual cortex regions with the strongest associations, before (*light bars*) and after controlling for age (*dark bars*). (**B**) presents a schematic of the visual cortex regions included in the analysis. (**C–H**) depict correlations for individual retinal layers: RNFL **C**, GCIPL **D**, INL **E**, OPL **F**, ONL **G**, and PR **H**. Statistical significance is indicated as *P* < 0.05 (*) and *P* < 0.01 (**). Most correlations were attenuated after adjusting for age, indicating age-mediated relationships. Notably, RNFL with Central Visual Striate 1 and INL with Peripheral Visual Inferior Extrastriate 2 remained significant after age adjustment, highlighting age-independent associations. This figure illustrates the layer-specific and region-specific relationships between retinal volumes and visual cortex structure in normal healthy aging.

RNFL volume was found to be related to cortical thickness in all 13 regions of the visual cortex, with the strongest correlations observed in the central visual striate 1, peripheral visual inferior extrastriate, two regions of the central visual extrastriate, and the peripheral visual striate calcarine (Fig. 4C). When controlling for age, the RNFL was only significantly related to the central visual striate (*P* < 0.01). Additionally, all five of these key brain regions showed a significantly negative correlation with age (*P* < 0.05; see Table 1).

Retinal volume in the GCIPL was associated with cortical thickness in all 13 regions of the visual cortex, with the strongest correlations found in two regions of the peripheral visual superior extrastriate, two regions of the peripheral visual inferior extrastriate, and the central visual extrastriate (Fig. 4D). However, after controlling for age, none of the regions showed a significant correlation with GCIPL. Additionally, all five regions exhibited a significant negative correlation with age (*P* < 0.05; see Table 1).

INL volume showed moderate correlations with five visual cortex regions (*P* < 0.05), including central and peripheral visual areas (Fig. 4E). These associations were attenuated after adjusting for age, with one correlation (peripheral visual inferior extrastriate 2) remaining significant (*P* < 0.05). Whereas INL volume itself was not significantly associated with age, all five cortical regions exhibited significant negative correlations with age (*P* < 0.05; see Table 1).

Retinal volumes in both the OPL and ONL were not significantly associated with cortical thickness in any regions of the visual cortex, as demonstrated by the top five correlation regions and their values (Figs. 4F, 4G). After controlling for age, these correlations further decreased for both layers.

Retinal volume in the PR layer showed no significant correlation with cortical thickness in any of the examined visual cortex regions, as illustrated by the top five correlation values (Fig. 4H). Although the unadjusted correlations were weakly negative, these associations became positive but remained nonsignificant after controlling for age. Each of these five brain regions exhibited a statistically significant negative correlation with age, whereas the PR layer was positively related to age (*P* < 0.01).

## Discussion

Whereas previous studies have shown that normal aging induces changes in the retina and brain,^30^^–^^35^ our study is the first to use quantitative structural MRI and OCT retinal segmentation together to demonstrate distinct age-mediated and age-independent relationships between specific retinal layers and the visual cortex. Although associations involving TRT, RNFL, and GCIPL were predominantly influenced by age, selected relationships involving the RNFL and INL were independent of age.

Whereas most correlations between retinal layers and cortical regions were attenuated after adjusting for age, the correlation between RNFL and Central Visual Striate 1 remained statistically significant. This finding suggests that RNFL thickness may reflect the structural integrity of the primary visual cortex independently of age, indicating a potentially direct anatomic and functional relationship between retinal ganglion cell axons and cortical architecture. The age-related changes observed in the brain are likely a result of neuronal volume loss,^30^^,^^34^ suggesting that alterations in TRT, RNFL, and GCIPL reflect underlying neuronal changes in the brain.

Therefore, the volume of TRT, RNFL, and GCIPL may serve as valuable surrogate markers for age-related neuronal changes in the visual cortex. Understanding the typical age-related changes can establish a baseline for distinguishing between retinal changes associated with normal aging and those indicative of disease.^31^ Furthermore, the INL showed no significant correlation with age but maintained an association with the visual cortex even after adjusting for age. This suggests that this relationship is not driven by normal aging and may instead reflect other underlying factors. Consequently, changes in INL volume could serve as potential indicators of disease processes specifically affecting the visual system.

Interestingly, unlike the age-related thinning observed in inner retinal layers such as the GCIPL and RNFL, the PR layer showed a positive correlation with age in the present study, which is in agreement with previous studies.^31^^,^^36^^,^^37^ This likely reflects unique age-related changes in the outer retina, including PR outer segment remodeling or accumulation of extracellular material like lipofuscin.^38^ These structural alterations differ from the neurodegenerative thinning seen in inner retinal layers and suggest that PR layer changes could represent compensatory or adaptive responses during aging.^31^ Recognizing these layer-specific differences may facilitate better understanding of the normal aging process.

Atrophy in the aging brain is thought to arise from several physiological mechanisms, including mitochondrial dysfunction, genomic instability, dysregulated nutrient sensing, loss of proteostasis, and the accumulation of reactive oxidative species.^39^ The retina also reflects this change, demonstrating an overall decrease in thickness with age.^30^^,^^40^^–^^42^ When controlling for age, TRT, RNFL, and GCIPL showed correlations with fewer regions of the visual cortex, suggesting that age is a primary mediator in the correlation between the brain and retina.

The TRT volume consists of six distinct layers. Among these, the ganglion cell complex is formed by the GCIPL and the RNFL, creating a complex network of neurons. The RNFL comprises axons, whereas the GCIPL contains these neurons’ cell bodies and dendrites.^43^ Given that a significant portion of TRT derives from these neuronal structures, it is plausible that the retina undergoes age-related changes that parallel those observed in the brain. Furthermore, retinal atrophy associated with aging may contribute to additional deterioration in the visual cortex.^44^

In this study, among the seven retinal layers examined, TRT, RNFL, and GCIPL volumes showed the strongest correlations with the visual cortex, and each demonstrated a negative correlation with age, consistent with previous research.^45^^,^^46^ As the thickest retinal layers, composed of neurons, the RNFL and GCIPL likely reflect the neuronal integrity in the brain, thus strengthening the connection between TRT and brain function. The more pronounced decline in volume observed in these neuronal layers compared with the other layers suggests that age-related reductions in retinal tissue volume may primarily stem from neurodegeneration rather than a general loss of overall tissue volume.

Whereas this study reveals an age-mediated correlation between the structure of the retina and the visual cortex, the INL maintained its association with the visual cortex even after controlling for age. This suggests that factors beyond age mediate the connection between the retina and the brain. Notably, retinal degeneration has been observed in various disease processes, including intracranial tumors.^47^ Namely, the GCIPL, RNFL, and INL demonstrate thinning in individuals with certain intracranial tumors through retrograde transsynaptic degeneration.^47^

Thus, whereas age is a primary mediator in the correlation between the brain and the retina, other factors must be considered and further explored. The INL is significantly thinner than the RNFL and GCIPL and thus does not contribute as significantly to the TRT changes with aging.^6^^,^^37^ The INL has been observed to develop atrophy and retrograde transsynaptic degeneration in disease states such as multiple sclerosis (MS) and optic chiasm damage.^48^ Because an association between the INL and visual cortex persists after adjustment for age, unlike the predominantly age-driven associations seen with TRT, RNFL, and GCIPL, structural changes in the INL may serve as more specific indicators of cortical disease rather than generalized aging effects.

The OPL and ONL were not significantly associated with any visual cortex regions, either before or after adjusting for age, suggesting a weaker structural link to cortical anatomy. This may reflect their anatomic roles; OPL contains synapses between PRs and bipolar cells, whereas the ONL houses PR nuclei, structures less directly involved in transmitting signals to the brain.^49^^,^^50^ These layers are also less affected by aging and neurodegeneration compared with inner retinal layers, supporting their limited utility as indicators of brain-related changes.

Recent work by Han et al. investigated associations between macular RNFL thickness and brain volume and cortical thickness in non-demented older adults. In line with our findings, their study identified several retina–brain associations at uncorrected thresholds that did not remain statistically significant after correction for multiple comparisons, highlighting modest effect sizes.^51^ Although Han et al. focused primarily on RNFL thickness in an older cohort and examined whole-brain associations, our study extends this literature by incorporating detailed intraretinal layer segmentation, region-specific analyses of the visual cortex, and explicit adjustment for age. Importantly, our results suggest that many retina–brain correlations are largely age-mediated, whereas the association between RNFL and the primary visual cortex persists independently of age, supporting a potentially more direct structural relationship within the visual system.

This study has limitations, including a relatively small sample size (*n* = 53) and the lack of a longitudinal follow-up with participants. Furthermore, our analysis did not explore how the decline in retinal volume or brain thickness over time affects cognition or visual function. Despite these constraints, we identified statistically significant correlations between the volume of specific retinal layers that correlate with age and individually mapped brain regions, extending the concept of the retina as a window to the brain. Retinal volume was chosen instead of thickness alone because it provides a more robust and spatially integrated measure across the perifoveal region, which helps account for anatomic variability around the foveal pit. Prior studies have shown that volume-based measurements improve the stability and sensitivity of retinal assessments, particularly when evaluating correlations with brain structure.^52^^,^^53^ On the other hand, for the brain, we used cortical thickness, derived via ANTs from high-resolution T1-weighted MRI and parcellated using the Schaefer 200-region atlas. Cortical thickness is widely considered more sensitive to age-related neuronal atrophy than cortical volume, which can be influenced by variability in surface area and gyrification.^9^^,^^54^ Moreover, thickness is less affected by intersubject differences in brain size, which is particularly important in studies with broad age ranges. Nevertheless, future studies with larger sample sizes, longitudinal designs, and volume to volume comparison are needed to comprehensively assess the interplay between retinal and cerebral changes and their impact on cognitive processes over time.

## Conclusions

This study demonstrates that distinct retinal layers exhibit differential associations with the visual cortex, with some relationships mediated by age and others independent of it. The strong, age-related correlations of TRT, RNFL, and GCIPL with visual cortex regions suggest these layers reflect global neurodegenerative changes, whereas the INL's consistent association regardless of age points to its potential as a marker for localized or disease-specific cortical pathology. By integrating structural MRI and OCT segmentation, this work supports retinal imaging as a valuable tool for studying brain structure and highlights the need for future research to explore these relationships in clinical populations and through longitudinal analysis.
